# Immunological Efficacy of Tenofovir Disproxil Fumarate-Containing Regimens in Patients With HIV-HBV Coinfection: A Systematic Review and Meta-Analysis

**DOI:** 10.3389/fphar.2019.01023

**Published:** 2019-09-12

**Authors:** Taiyi Jiang, Bin Su, Ting Song, Zhiqiang Zhu, Wei Xia, Lili Dai, Wen Wang, Tong Zhang, Hao Wu

**Affiliations:** ^1^Center for Infectious Diseases, Beijing Youan Hospital, Capital Medical University, Beijing, China; ^2^Beijing Key Laboratory for HIV/AIDS Research, Beijing, China; ^3^Department of Urology, Beijing Youan Hospital, Capital Medical University, Beijing, China

**Keywords:** tenofovir disproxil fumarate, drug treatment, outcomes research, meta-analysis, HIV, hepatitis B virus, coinfection

## Abstract

**Background:** Hepatitis B virus (HBV) coinfection is common in HIV-positive patients. HIV infection modifies the natural course of HBV infection, leading to a faster progression of liver-related morbidity and mortality than is observed in HBV mono-infected patients. This systematic review and meta-analysis evaluates the current clinical evidence regarding the use of oral tenofovir disproxil fumarate (TDF)-based treatments in patients coinfected with HIV and HBV.

**Methods:** We performed a comprehensive literature search in PubMed and Web of Science. Supplementary searches were conducted in Google Scholar and Clinicaltrials.gov. We conducted a random effects meta-analysis using the event rate (ER) to estimate the incidence of HBV seroconversion. A subgroup meta-analysis was performed to assess the moderate effects of demographic and disease-related variables on HBsAg loss. This review is registered in the PROSPERO database (CRD42018092379).

**Results:** We included 11 studies in the review. The immunological effects of oral TDF-based Pre-exposure prophylaxis (PrEP) treatment in patients with HIV-HBV coinfection were 0.249 for HBeAg loss, 0.237 for HBeAg conversion, 0.073 for HBsAg loss, and 0.055 for HBsAg conversion. The factors associated with HBsAg loss were the baseline HBV viral load, participant’s location, and a history of exposure to lamivudine/emtricitabine (3TC/FTC) (all *p* < 0.05). A trend toward a negative relationship between the baseline CD4^+^ T-cell count and HBsAg loss was observed (*p* = 0.078).

**Conclusion:** This systematic review and meta-analysis demonstrated that TDF-containing regimens are effective at stimulating HBeAg loss (24.9%), HBeAg conversion (23.7%), HBsAg loss (7.3%), and HBsAg conversion (5.5%) in HIV-HBV coinfected patients. The moderator analysis showed that HBV viral load, the location of participants, and prior exposure to 3TC/FTC are factors associated with HBsAg loss. Asian ethnicity, prior exposure to 3TC, and a nondetectable baseline HBV viral load are associated with lower odds of HBsAg loss. Well-designed prospective cohort studies and randomized controlled trials (RCTs) with large sample sizes are required for the investigation of potential predictors and biological markers associated with strategies for achieving HBV remission in patients with HIV-HBV coinfection, which is a matter of considerable importance to clinicians and those responsible for health policies.

## Introduction

Approximately 5–25% of acquired immunodeficiency syndrome (AIDS) patients are coinfected with hepatitis B virus (HBV) (Unaids, 2018). Human immunodeficiency virus (HIV) infection modifies the natural course of HBV infection, leading to a faster progression of liver-related morbidity and mortality than is observed in HBV mono-infected individuals, accompanied by a higher prevalence of antiretroviral therapy (ART)-related hepatotoxicity (Avihingsanon et al., 2010). Recent studies have reported that liver disease continues to progress in 10–20% of individuals on tenofovir-containing HBV-active ART (Coffin et al., 2013; Vinikoor et al., 2017). Tenofovir disproxil fumarate (TDF) is one of the most commonly/widely used nucleotide reverse transcriptase inhibitors (NRTI) for the treatment of HIV and HBV and is recommended by the current HIV treatment guidelines (Aidsinfo, 2018). TDF-containing regimens are particularly favored for the clinical treatment of HIV-HBV coinfection in areas in which resources are limited.

Tenofovir is widely used as a first-line agent for the treatment of chronic HBV infection due to the relatively low levels of drug resistance and high virological efficacy (Nunez et al., 2002; Ristig et al., 2002; Dore et al., 2004; Bihl et al., 2015). A recent study showed that TDF treatment resulted in undetectable levels of HBV in approximately 90% of patients with HIV-HBV coinfection. This proportion increased rapidly over the first 2 years of treatment and continued to rise slowly thereafter (Price et al., 2013). Moreover, there is currently no confirmed evidence of mutations conferring resistance to TDF in the HBV strains harbored by these patients (Kitrinos et al., 2014).

Interest has recently focused on trying to cure chronic HBV infection. NRTI treatment has been shown to decrease the formation of stable episomal covalently closed circular DNA (cccDNA) and, to a lesser extent, the integration of HBV DNA into the host genome, but HBsAg continues to be produced. Sustained high levels of HBsAg have been associated with a high risk of hepatocellular carcinoma (HCC) in cases of untreated HBV mono-infection (Tseng et al., 2012; Yang et al., 2016). The persistence of cccDNA and HBsAg are the main barriers to curing HBV (Zeisel et al., 2015). HBsAg seroclearance and the development of antibodies against HBsAg can be used to assess HBV function.

Prolonged periods of good response to TDF treatment have been achieved, with HBV DNA remaining undetectable in the serum, but the elimination of cccDNA is the ultimate goal in strategies that aim to cure HBV infection. The concept of a functional cure, defined as the clearance of HBsAg or persistent seroconversion during treatment that may also improve clinical outcomes, has recently been proposed. A loss of the HBsAg biomarker and seroconversion are clearly associated with lower levels of viral activity in the liver and the achievement of HBV remission. The hepatitis B “e” antigen (HBeAg) can also be used as an alternative biomarker of clinical remission, providing another endpoint indicating a long-term response to NRTIs.

Various studies have shown/reported various degrees of HBsAg loss and/or different seroconversion rates but found differences between coinfections and mono-infections, with higher rates in cases of coinfection. Cumulative HBsAg seroclearance rates of 5% to 22% have been reported (Jaroszewicz et al., 2012; Kosi et al., 2012; Maylin et al., 2012; Zoutendijk et al., 2012; Hamers et al., 2013; Matthews et al., 2013; Boyd et al., 2015; Huang and Nunez, 2015; Boyd et al., 2016; Lucifora and Protzer, 2016; Price et al., 2017). A recent meta-analysis focused exclusively on the suppression of HBV with TDF-containing ART (Price et al., 2013). However, there have been few descriptions of HBeAg loss or seroconversion to anti-HBe, HBsAg loss, and the adverse effects of long-term treatment. Therefore, we performed this meta-analysis on data from patients with HIV/HBV coinfection to confirm the utility of HBsAg and HBeAg loss rates as biomarkers and to assess the seroconversion rates and determinants of HBsAg seroclearance during TDF-based treatment for the long-term follow-up of patients coinfected with HIV and HBV. We also considered the factors affecting HBsAg loss.

## Methods

This systematic review and meta-analysis were performed in accordance with PRISMA guidelines (Moher et al., 2009a; Moher et al., 2009b; Moher et al., 2009c), and the study is registered in the International Prospective Register of Systematic Reviews (PROSPERO, https://www.crd.york.ac.uk/PROSPERO/): CRD42018092379. The PRISMA checklist is included in **Supplementary Table S1**.

### Search Strategy

A comprehensive literature search was performed in PubMed and Web of Science. The search terms used were intersections of treatment-related terms (TDF OR tenofovir) and disease terms (HIV OR AIDS OR HBV). Additional searches were also conducted in Google Scholar and ClinicalTrials.gov.

### Selection Criteria

**Figure 1:**
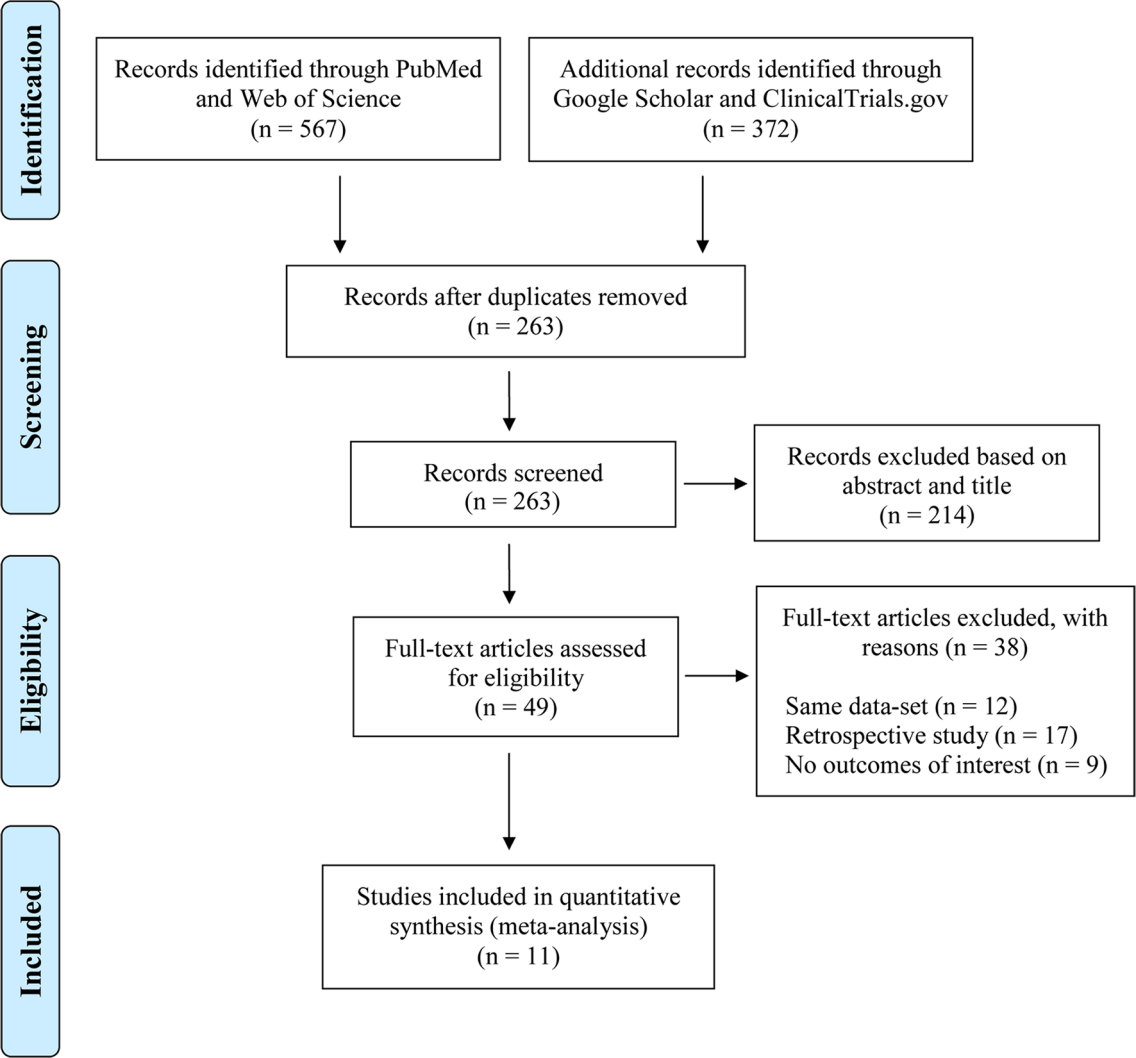
Flow chart of included studies.

The inclusion criteria were as follows: 1) The study design had to be a randomized controlled trial (RCT) or prospective cohort study; 2) the treatment regimen had to contain TDF with or without lamivudine (3TC) and/or emtricitabine (FTC); and 3) there had to be more than 10 participants in the TDF arm to prevent participant bias. We excluded 1) case reports; 2) review articles or theoretical articles; and 3) PhD theses, dissertations, and book chapters. Thus, to be more specific, 1) the participants were HIV-HBV coinfected patients at the screening stage of each study; 2) the eligible intervention contained TDF with or without 3TC and/or FTC, which are commonly used treatment combinations in most countries and regions; 3) some studies included in our meta-analysis were single-arm observational cohorts, while some studies compared the effectiveness between different treatment regimens; if the study arms used the medication combinations of interest, we included all arms; 4) because we aimed to investigate the immunological effects of targeted treatments, the outcomes of interest were HBV-related physiological processes during treatment. HBeAg and HBsAg are two key biomarkers for these processes; thus, we shifted our attention to the micro-level to detect the potential treatment efficacy. Two researchers independently performed the initial search, selecting studies on the basis of their titles and abstracts. The studies retained were then independently screened by a full-text assessment performed by the same researchers (TJ and TS). Disagreements between reviewers about study eligibility were resolved by discussion with BS. The procedure used for this study selection and the numbers of studies included and excluded are shown in **Figure 1**.

### Data Extraction and Code

The data of interest were independently extracted by two researchers (TJ and WX). The outcomes of interest were HBsAg and HBeAg seroconversion. The additional information extracted from articles included article author(s), year of publication, study location, sample size, study design, treatment regimen, and disease-related variables.

## Data Analysis

We performed a quantitative analysis with Comprehensive Meta-Analysis (CMA) Version 2.0 (Biostat, Englewood, NJ, U.S.). We first calculated the combined event rate (ER) from the number of events and the sample size of the TDF-based treatment arm. A random effects meta-analysis was conducted with the ER to estimate HBeAg loss, HBeAg conversion, HBsAg loss, and HBsAg conversion. Thus, the ER in different figures denotes the pooled ERs for HBeAg loss, HBeAg conversion, HBsAg loss, and HBsAg conversion. The variation in effect size across studies was assessed by calculating the homogeneity statistic Q. The I^2^ statistic was also used to estimate the proportion of heterogeneity in the observed variance (Higgins and Thompson, 2002). A subgroup meta-analysis was performed to assess the moderate effects of demographic and disease-related variables on HBsAg loss.

### Study Quality and Publication Bias

The Newcastle-Ottawa Scale (NOS) was adopted to evaluate the study quality of the nonrandomized studies, and the Physiotherapy Evidence Database (PEDro) scale was used to assess the study quality of RCTs (http://www.ohri.ca/programs/clinical_epidemiology/oxford.asp) (Moseley et al., 2002). The individual study quality for the included studies is shown in **Supplementary Table S2**.

Egger’s intercept test and fail-safe N were used to assess publication bias across studies (Rosenthal, 1979; Stuck et al., 1998). The trim-and-fill method was used if significant publication bias was detected by Egger’s test (Rosenthal, 1979). A subgroup meta-analysis was performed to assess the moderate effects of demographic and disease-related variables on HBsAg loss.

## Results

### Characteristics of the Studies Included

We identified 11 studies eligible for this review, with sample sizes ranging from 10 to 100 (Dore et al., 2004; Stephan et al., 2005; Matthews et al., 2008; Nuesch et al., 2008; Avihingsanon et al., 2010; Hamers et al., 2013; Matthews et al., 2013; Huang and Nunez, 2015; Boyd et al., 2016; Li et al., 2016; Wu et al., 2016). We included three RCTs and eight prospective cohort studies. All participants were adults over the age of 18 years. The most commonly/frequently used ART regimen was TDF with 3TC or FTC. Five studies examined TDF-naive patients, whereas the other six studies examined treatment in 3TC-experienced patients. These studies reported various outcomes, including seroconversion for HBsAg and HBeAg. Detailed information about the studies included is provided in **Table 1**.

**Table 1:** Characteristics of the included studies.

Names	Location	Year of Publication	N	Study Design	Treatment Regimen	Baseline HBV RNA (log10 c/ml)	Baseline HIV RNA (log10 c/ml)	CD4 (cells/μl)	Duration (weeks)
Matthews	Thailand	2008	23	RCT	TDF+3TC^@^ TDF^@^	8.4 8.6	4.7 5	39 25	48 48
Li	China	2016	91	Prospective	TDF+3TC ^#^	3.49	4.7	229	48
Stephan	German	2005	31	Prospective	TDF-based^#^				48
Matthews	Thailand	2013	47	Prospective	3TC+TDF or TDF/FTC^@^	8.56	4.71	48	108
Dore	Western Europe, North America, Australia	2004	10	Prospective	TDF-based^#^	8.6	3.4	497	48
Wu	China	2016	100	Prospective	TDF+3TC^@#^	6.9	4.2	186.5	48
Huang	Taiwan	2016	89	Prospective	TDF-based^#^	6	4.7	361	144
Nuesch	Thailand	2008	16	RCT	TDF/FTC^@^	4.6	2.7	363	69
Avihingsanon	Netherlands Australia Thailand	2010	10	RCT	TDF/FTC^@^	8.54	4.9	69	48
Boyd	Côte d’Ivoire South Africa	2016	85	Prospective	TDF/FTC^@^				142
Hamers	South Africa, Zambia	2013	93	Prospective	TDF-based^@#^	5.18	4.91		48

^@^TDF-naïve; ^#^3TC-experience.

### HBeAg Loss

**Figure 2:**
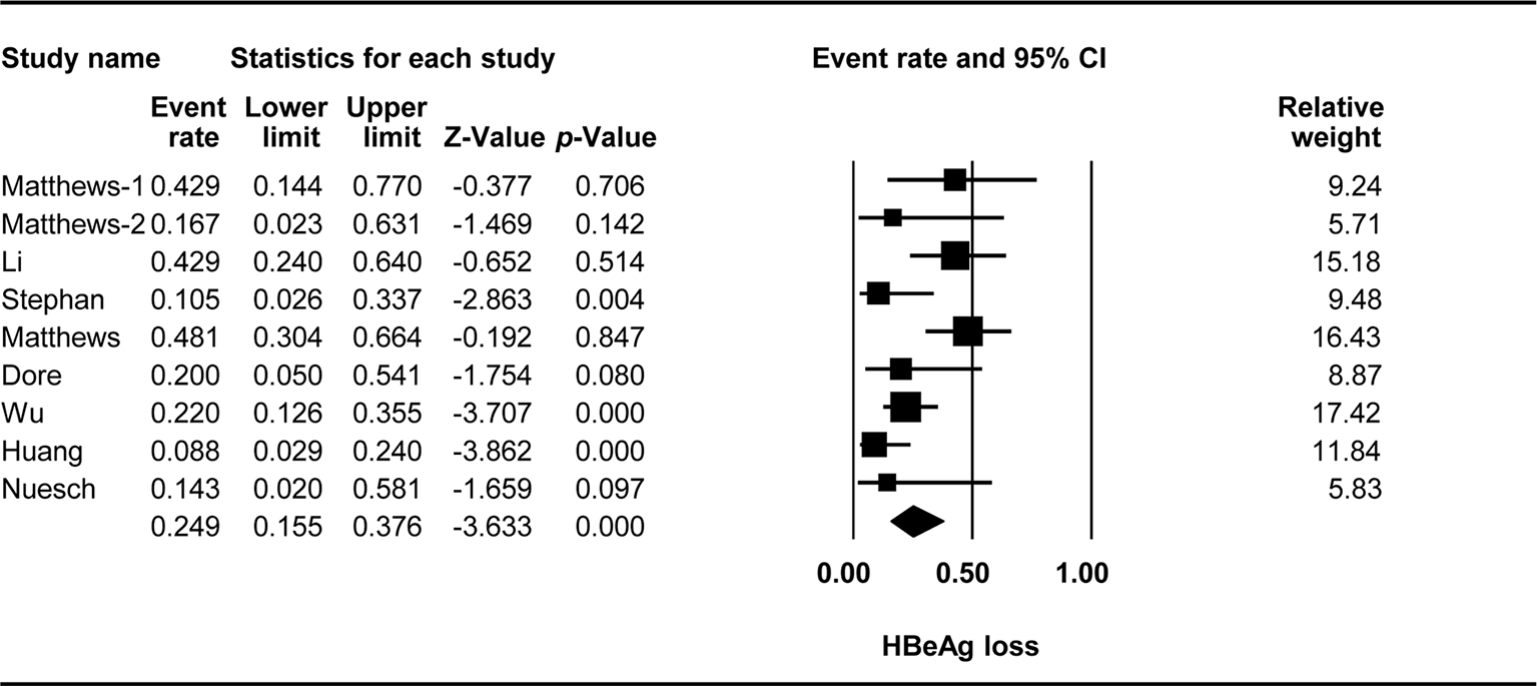
Forest plot for HBeAg loss rates during TDF-containing treatment.

The effect of TDF-containing treatment on HBeAg loss was reported for nine arms in eight studies. Therefore, it was possible to analyze the ER of each study. Egger’s intercept test showed no significant publication bias (Kendall’s tau = −1.556, *p* = 0.264), and the classic fail-safe N test showed that 63 missing studies would be required to obtain a non-significant result (*p* > 0.05). The combined ER for HBeAg loss was 0.249 (95% CI: 0.155–0.376, *p* < 0.001, **Figure 2**). There was significant heterogeneity across studies [Q(8) = 18.092, *p* = 0.021, I^2^ = 55.782].

### HBeAg Conversion

**Figure 3:**
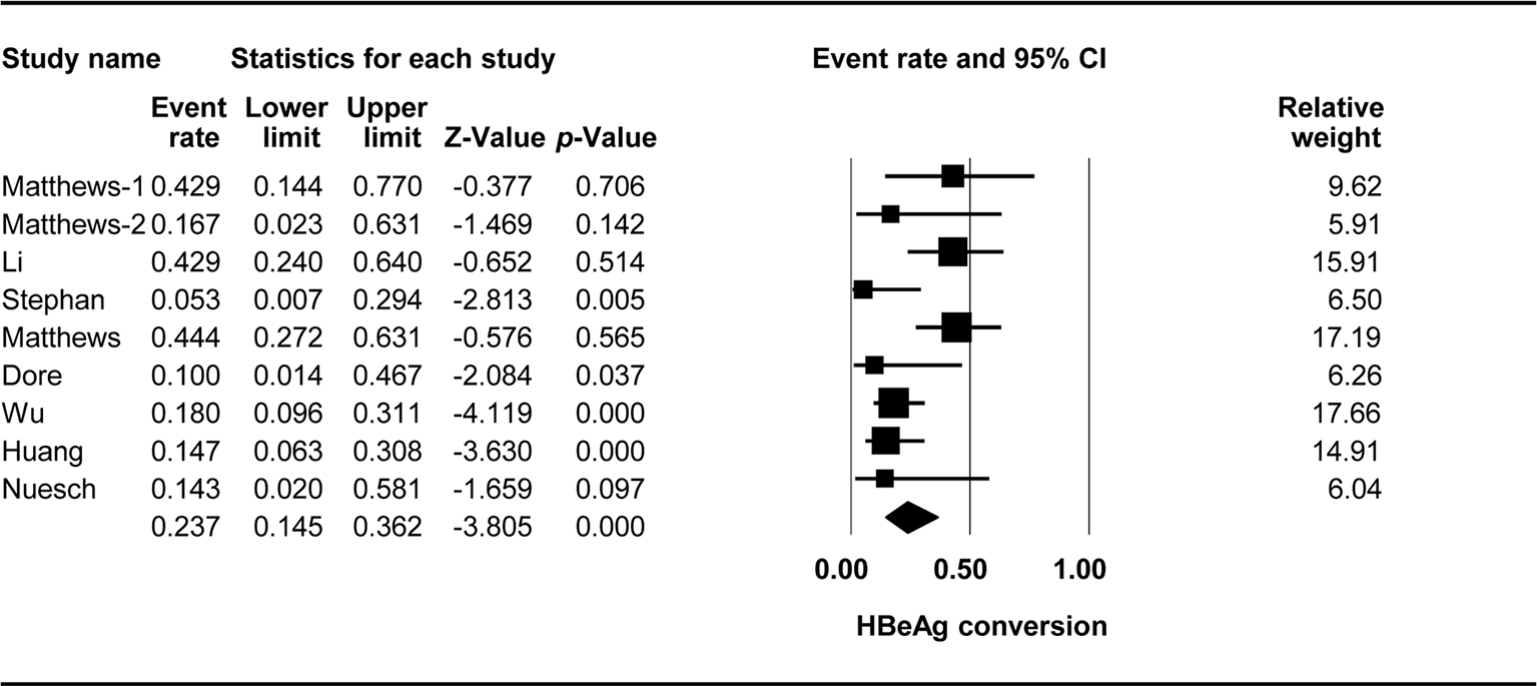
Forest plot for HBeAg conversion rates during TDF-containing treatment.

The effect of TDF-containing treatment on HBeAg conversion was reported for nine arms in eight studies, making it possible to analyze the ER of each of these studies. Egger’s intercept test showed that there was no significant publication bias (Kendall’s tau = −1.461, *p* = 0.127), and the classic fail-safe N tests showed that 70 missing studies would be required to obtain a non-significant result (*p* > 0.05). The combined ER for HBeAg loss was 0.237 (95% CI: 0.145–0.362, *p* < *0*.001, **Figure 3**). There was significant heterogeneity across studies [Q(8) = 17.405, *p* = 0.026, I^2^ = 54.036].

### HBsAg Loss

**Figure 4:**
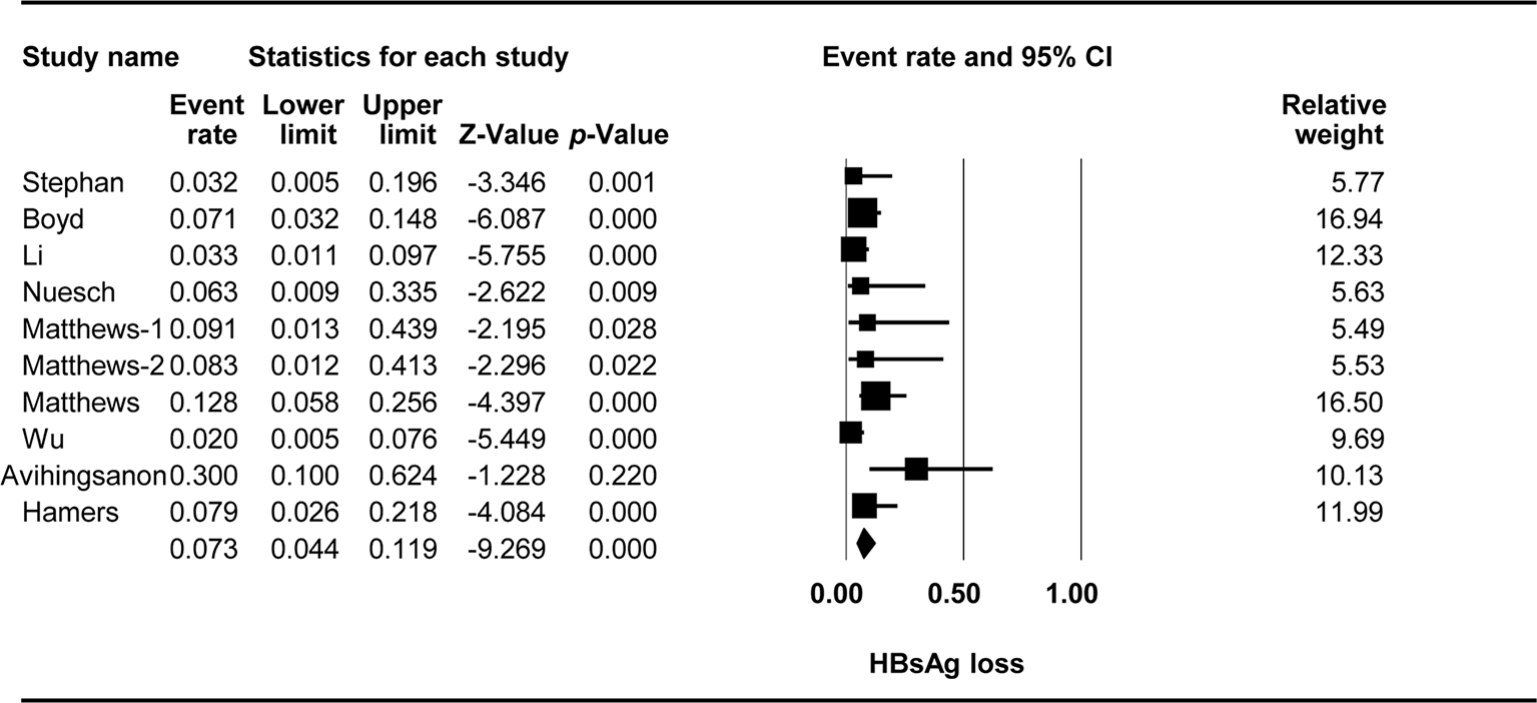
Forest plot for HBsAg loss rates during TDF-containing treatment.

The effect of TDF-containing treatment on HBsAg loss was reported for 10 arms in nine studies, so it was possible to analyze the ER of each study. Egger’s intercept test showed that there was no significant publication bias (Kendall’s tau = -0.667, *p* = 0.617), and the classic fail-safe N test showed that 356 missing studies would be required to obtain a non-significant result (*p* > 0.05). The combined ER for HBsAg loss was 0.073 (95% CI: 0.044–0.119, *p* < *0*.001, **Figure 4**). There was no significant heterogeneity across studies [Q(9) = 14.433, *p* = 0.108, I^2^ = 37.641].

Three studies with four arms were performed in Asia, and the other six studies were performed elsewhere. Stratification based on the location showed that location had a significant effect on the TDF-containing regimens [Q(1) = 5.233, *p* = 0.022, Asia vs. other countries: 0.037 (95% CI: 0.018–0.077) vs. 0.099 (95% CI: 0.058–0.164)].

Two studies included patients with CD4^+^ T-cell counts of at least 200 cells/µl, whereas five studies included patients with fewer than 200 cells/µl. Stratification based on the baseline CD4^+^ T-cell counts revealed an effect of marginal significance on the efficacy of TDF-containing regimens [Q(1) = 3.095, *p* = 0.078, 200 or more cells/µl vs. fewer than 200 cells/µl: 0.039 95% CI: 0.015–0.098 vs. 0.094 95% CI (0.026–0.218)].

In five studies with six arms, 3TC was used before TDF, whereas in three studies, TDF was used in patients not previously exposed to 3TC. Prior exposure to 3TC significantly affected the efficacy of TDF-containing regimens [Q(1) = 4.204, *p* = 0.04, yes vs. no: 0.041 95% CI (0.017-0.099) vs. 0.109 95% CI (0.068–0.169)].

Six studies reported detectable HBV at the baseline, whereas three studies with four arms reported undetectable HBV at the baseline. The baseline HBV viral load significantly affected the efficacy of TDF-containing regimens [Q(1) = 7.938, *p* = 0.005, yes vs. no: 0.147 95% CI (0.083–0.247) vs. 0.05 95% CI (0.031–0.08)].

### HBsAg Conversion

**Figure 5:**
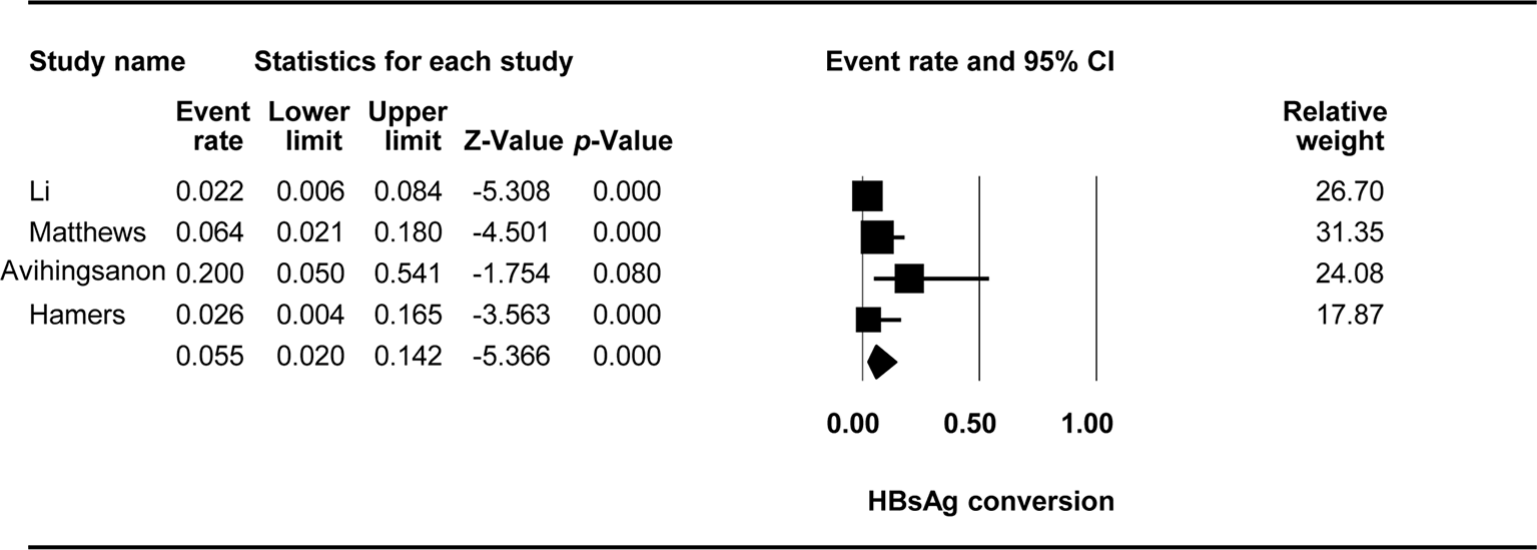
Forest plot for HBsAg conversion rates during TDF-containing treatment.

The effect of TDF-containing treatments on HBsAg loss was reported in nine studies with 10 arms, making it possible to analyze the ER of each of these studies. Egger’s intercept test showed that there was no significant publication bias (Kendall’s tau = −0.753, *p* = 0.886), and the classic fail-safe N test showed that 56 missing studies would be required to obtain a non-significant result (*p* > 0.05). The combined ER for HBeAg loss was 0.055 (95% CI: 0.02–0.142, *p* < *0*.001, **Figure 5**). There was no significant heterogeneity across studies [Q(3) = 5.81, *p* = 0.121, I^2^ = 48.365].

## Discussion

This systematic review and meta-analysis provides pooled estimates of the serological outcomes of TDF-containing regimens in patients with HIV-HBV coinfection. The overall estimates are useful for targeted treatments in key populations.

We found that almost a quarter of the participants experienced serological changes in HBeAg. However, these results should be interpreted with caution due to a relatively high degree of heterogeneity among the studies included. The rates of HBeAg loss ranged from 8.8% to 48%, and the rates of HBeAg seroconversion ranged from 10% to 44%. The causes of this heterogeneity should be identified, as long-term treatment resulted in higher rates of HBeAg loss and seroconversion. The combination of TDF + 3TC/FTC seemed to be more effective in patients with documented 3TC resistance (Luo et al., 2018). Differences in immune restoration after ART initiation were observed, with various rates of HBeAg seroclearance, as a sudden increase in CD4^+^ T-cell counts may promote a rapid immune response (Miailhes et al., 2007).

HBsAg loss was observed in 7.3% of the coinfected patients, and the HBV curve was recorded in 5.5% of participants, which was consistent with the results of previous observational studies (Martin-Carbonero et al., 2011; Van Griensven et al., 2014). Understanding the predictors of HBsAg loss is an important research priority in the search for novel strategies for achieving HBV remission, and individuals with HIV-HBV coinfection may constitute a unique group for studying such associations.

Minor immunosuppression appeared to influence the baseline HBsAg and HBeAg levels, with a negative impact on the decrease in HBsAg and HBeAg levels. In our review, we found that patients with CD4^+^ T-cell counts below 200 cells/μl and higher HBV DNA levels at the baseline were more likely to display HBsAg loss than were their counterparts. This might reflect robust immune reconstitution and the acquisition of enhanced pathogen-specific innate or adaptive immune responses following treatment with TDF-containing regimens (Hsu et al., 2012; Boyd et al., 2015). However, a previous study showed that patients with CD4^+^ T-cell counts below 300 cells/μl during TDF-containing treatment, a level considered to constitute mild immunosuppression, cannot achieve a strong enough immune response to clear infected hepatocytes (De Vries-Sluijs et al., 2010).

Higher levels of HBV DNA and exposure to 3TC are associated with longer times before the achievement of undetectable levels of HBV DNA while receiving TDF (Childs et al., 2013), and a longer time to the occurrence of an immunological response represented by HBsAg loss. Therefore, studies in real-world settings are required to determine the effects on HBsAg and HBeAg seroclearance in patients with prior 3TC treatment and patients receiving TDF-based ART as the initial treatment.

We detected differences between Asia and other parts of the world, with HIV-induced immunosuppression associated with different degrees of HBsAg loss in patients with HBV-HIV coinfection. Many studies have shown that genotype A is the most prevalent HBV genotype in non-Asian populations, which display higher levels of HBeAg and HBsAg during natural infections and following IFN treatment (Erhardt et al., 2005; Flink et al., 2006; Thio and Locarnini, 2007). The HBV genotype distribution is different in Asia and other regions; thus, the subtypes of HBV and their virological responses require further investigation.

Although the safety of TDF-containing regimens was not our primary outcome of interest in this meta-analysis, substantial attention should be paid by health providers and policymakers to monitoring potential side effects in people living with HIV-HBV coinfection. An elevated incidence of renal and liver dysfunction was detected among HIV-HBV coinfected participants on long-term TDF treatment in recent studies (Peters et al., 2006; Tan et al., 2009). Although few studies have reported potential side effects on bone mineral density among HIV-HBV coinfected patents, a recent systematic review and meta-analysis among people mono-infected with HBV or HIV have reported reduced bone density and an elevated incidence of bone fracture due to long-term use of TDF-containing treatments (Buti et al., 2018; Goh et al., 2018). Thus, considering the high availability and low cost of TDF-based treatment in middle- or low-income settings among HIV-HBV coinfected participants, it is better to closely monitor and promptly treat these side effects. In addition, in developed or high-income countries, clinicians may choose treatment regimens with fewer side effects, such as tenofovir alafenamide (TAF)-based treatment regimens.

This review has several limitations. First, the results should be interpreted with caution due to the limited number of comparisons included, potentially restricting the external validity of the conclusions in other settings and decreasing the statistical power to detect potentially significant results. Second, the relatively high level of heterogeneity may have decreased the representativeness of some outcomes (i.e., HBeAg loss and conversion). Third, only a few studies reported adverse events related to kidney and liver function, and this may limit attempts to quantitatively assess the safety of TDF-containing regimens in patients with HIV-HBV coinfection.

## Conclusion

This systematic review and meta-analysis demonstrated that TDF-containing regimens are effective at stimulating HBeAg loss (24.9%), HBeAg conversion (23.7%), HBsAg loss (7.3%), and HBsAg conversion (5.5%) in HIV-HBV coinfected patients. The moderator analysis showed that Asian ethnicity, prior exposure to 3TC, and nondetectable baseline HBV viral load are associated with lower odds of HBsAg loss. However, we should cautiously interpret the results regarding HBeAg loss and HBeAg conversion due to significant heterogeneity across all study arms. Well-designed prospective cohort studies and RCTs with large sample sizes are required for the investigation of potential predictors and biological markers associated with strategies for achieving HBV remission in patients with HIV-HBV coinfection, which is a matter of considerable importance to clinicians and those responsible for health policies.

## Author Contributions

TJ, BS, and HW conceived and designed the protocol and study. TJ, ZZ, WX, TZ, and HW identified studies to be screened. TJ, BS, TS, LD, WW, and TZ identified studies for eligibility, extracted data, and assessed the methodological quality of included studies. TJ and BS wrote the manuscript. All authors read and approved the final manuscript.

## Funding

This work was supported by the National 13th Five-Year Grand Program on Key Infectious Disease Control (2018ZX10301-407-005 and 2018ZX10302103-001-003 to TJ, 2017ZX10202102-005-003 to BS, and 2017ZX10202101-004-001 to TZ), the National Natural Science Foundation of China (NSFC, 81772165 to BS and 81571973 to HW), the NSFC-NIH Biomedical collaborative research program (81761128001 to HW), the Beijing Municipal of Science and Technology Major Project (D161100000416003 to HW), the Funding for Chinese overseas talents returning to China in 2016-Beijing Municipal Human Resources and Social Security Bureau (to BS), and the Beijing Key Laboratory for HIV/AIDS Research (BZ0089). The funders had no role in the study design, data collection and analysis, decision to publish, or preparation of the manuscript.

## Conflict of Interest Statement

The authors declare that the research was conducted in the absence of any commercial or financial relationships that could be construed as a potential conflict of interest.
