# Supplementary material for: Immunological Efficacy of Tenofovir Disproxil Fumarate-Containing Regimens in Patients With HIV-HBV Coinfection: A Systematic Review and Meta-Analysis
Source: Front Pharmacol. 2019 Sep 12;10:1023. doi: 10.3389/fphar.2019.01023 (PMC6752181; doi:10.3389/fphar.2019.01023)
Supplement: Supplementary file 2 [file Table_2.docx]

**Table S2 Study quality for individual studies stratified by study design**

| Name | Study design | NOS | PEDro |
| --- | --- | --- | --- |
| Matthews (2008) | RCT |  | 9 |
| Li (2016) | Prospective | 7 |  |
| Stephan (2005) | Prospective | 6 |  |
| Matthews (2013) | Prospective | 6 |  |
| Dore (2004) | Prospective | 7 |  |
| Wu (2016) | Prospective | 9 |  |
| Huang (2016) | Prospective | 7 |  |
| Nuesch (2008) | RCT |  | 7 |
| Avihingsanon (2010) | RCT |  | 9 |
| Boyd (2016) | Prospective | 7 |  |
| Hamers (2013) | Prospective | 5 |  |

NOS=Newcastle-Ottawa Scale; PEDro=Physiotherapy Evidence Database
